# Juvenile dermatomyositis in Oman: clinical patterns and disease trajectory from a National cohort

**DOI:** 10.1186/s12969-025-01132-0

**Published:** 2025-07-17

**Authors:** Reem Abdwani, Mahadev J. Mal, Eman Al Masroori, Ruqaiya Al Jashmi, Safiya Al Abrawi, Ibrahim Al-Zakwani

**Affiliations:** 1 Department of Child Health, College of Medicine & Health Science, Sultan Qaboos University, Muscat, Oman; 2 Department of Child Health, Sultan Qaboos University Hospital, University Medical City, Muscat, Oman; 3 Department of Child Health, Royal Hospital, Muscat, Oman; 4 Department of Pharmacology, College of Medicine and Health Sciences, Sultan Qaboos University Hospital, Muscat, Oman

**Keywords:** Juvenile dermatomyositis, Oman, Clinical presentation, Treatment outcome

## Abstract

**Objective:**

Juvenile dermatomyositis (JDM) is an uncommon autoimmune condition in children, often leading to prolonged disease burden and significant morbidity. Despite global advancements in understanding JDM, studies from the Middle East, particularly Oman, remain scarce. This study aims to characterize JDM from an Omani national cohort, evaluating clinical manifestations, laboratory features, disease course, and treatment outcomes.

**Methods:**

A retrospective review of all JDM patients diagnosed and managed by pediatric rheumatologist in tertiary centers in Oman was conducted. Patient demographics, clinical features, laboratory findings, treatment modalities, and disease outcomes were analyzed.

**Results:**

A total of 30 children diagnosed with JDM were included. They had an equal female to male distribution, 1:1 ratio. The median age at disease onset was 6.78 years (range: 2–13), with a median diagnostic delay of 8.4 months (range:1–23). The median follow-up period for these patients was 4 years (absolute range: 1 month-16 years). Classic JDM skin manifestations, including heliotrope rash (*n* = 25; 83%) and Gottron’s papules (*n* = 23; 77%), were common. Proximal muscle weakness was observed in 28 (93%) patients, while 23 (77%) patients exhibited elevated muscle enzymes. MRI findings consistent with myositis were present in 70% (*n* = 19/27) of the subjects, and muscle biopsy confirmed JDM in 9 cases (30%). Among 25 patients tested for myositis specific antibodies, NXP2 (*n* = 3), Anti-TIF1 (*n* = 2), Anti-Mi-2 (*n* = 1), and MDA5 (*n* = 1) were detected, showing expected correlations with disease phenotype. Corticosteroids were universally administered, with methotrexate (*n* = 25; 83%) and IVIG (*n* = 15; 50%) as common adjuncts. Calcinosis was observed in 8 patients (27%), and was managed with various treatment modalities including pamidronate (*n* = 3), diltiazem (*n* = 2), and infliximab (*n* = 1). At the last follow-up, 18 patients (60%) were in clinical remission, 50% (*n* = 15) followed a polyphasic or chronic disease course, and 2 patients succumbed to disease-related complications.

**Conclusions:**

This study provides comprehensive characterization of pediatric JDM in Oman. The findings highlight regional variations in disease presentation, autoantibody profiles, and treatment responses, underscoring the need for early diagnosis and individualized management strategies. Continued follow-up is essential to optimize long-term outcomes and improve survival rates in this patient population.

## Introduction

Juvenile dermatomyositis (JDM) is a rare systemic autoimmune disease with an estimated incidence of 2–4 cases per million children annually [1]. However, it is the most common subtype of juvenile idiopathic inflammatory myopathies (JIIM), accounting for about 85% of all cases [1]. The pathogenesis of JDM is uncertain, but it is likely the result from a complex interplay between infectious and environmental triggers in genetically susceptible individuals [2–6].

JDM is characterized by proximal muscle weakness and distinct cutaneous manifestations, including the heliotrope rash and Gottron’s papules. In addition to these hallmark features, systemic complications such as interstitial lung disease (ILD), cardiac dysfunction, and gastrointestinal vasculopathy can be observed, contributing to potential morbidity and mortality [7, 8]. Secondary malignancies are rarely associated with JDM, distinguishing them from adult-onset myositis [9].

The Bohan and Peter criteria for diagnosing JDM, developed over 40 years ago, remain widely used despite advancements in immunological and histopathological understanding [10]. Recent validation of these criteria through the International Myositis Classification Criteria Project (IMCCP) led to the development of new EULAR/ACR classification criteria, which demonstrate improved sensitivity, specificity, and diagnostic accuracy for both juvenile-and adult-onset idiopathic inflammatory myositis (IIM) [11].

The clinical presentation, prevalence, and outcomes of JDM exhibit considerable variability across populations. Studies from Saudi Arabia have reported higher incidence of JDM in male children [12]. Similarly, a cohort from Western India demonstrated a higher frequency of monocyclic disease course compared to cohorts in Europe and North America, where chronic or polycyclic courses are more prevalent​ [13]. Additionally, minority populations with lower socioeconomics are more likely to experience worse physical function, increased disease activity, and lower quality of life scores​ [14].

Despite global advancements in understanding JDM, studies and clinical data from the Middle East, particularly Oman, remain scarce [15–17]. This study aims to characterize JDM in a cohort of pediatric patients from Oman by examining the prevalence and spectrum of clinical manifestations, organ involvement patterns, autoantibody profiles, outcomes and associated comorbidities. The findings will provide population-specific insights to improve clinical awareness, facilitate early diagnosis, and guide tailored treatment approaches for JDM in Omani pediatric patients, while contributing to the global understanding of disease variability and regional differences.

## Methods

We conducted a retrospective observational study to evaluate the clinical characteristics, treatment patterns, and outcomes of patients diagnosed with JDM at tertiary care centers in Oman. The study included patients seen between January 2008 and December 2024 at major tertiary hospitals in Muscat, Sultan Qaboos University Hospital and Royal Hospital, Muscat, Oman, which serve as the primary referral centers for Pediatric Rheumatology in the country that cater to a diverse population, including Omani nationals and expatriates.

The study included patients, aged 13 years or younger, who met the Bohan and Peter classification criteria for probable or definite JDM. Patients were excluded if they did not meet the Bohan and Peter criteria [10] for probable or definite for JDM, were older than 13 years at diagnosis—as this exceeded the pediatric age limit at our institutions—or had incomplete medical records that prevented adequate longitudinal assessment. Data were extracted from electronic medical records, including demographic information, clinical features, laboratory results, electromyography (EMG), muscle biopsy, magnetic resonance imaging (MRI), treatment details, and outcomes.

Laboratory investigations included assessment of muscle enzymes, specifically creatine kinase (CK), lactate dehydrogenase (LDH), aspartate aminotransferase (AST), and alanine aminotransferase (ALT). Myositis-specific antibodies (MSA) and myositis-associated antibodies (MAA) were analyzed using the EUROLINE Myositis Profile immunoblot assay (Euroimmun, Germany). Results were interpreted semi-quantitatively in accordance with the manufacturer’s guidelines, with band intensities graded as 1+ (weak positivity), 2+ (moderate positivity), and 3 + or higher (strong positivity).

The primary outcomes were [1] clinical characteristics, diagnostic features, treatment patterns [2] disease progression over the follow-up period which was categorized as monocyclic, polycyclic and chronic remitting disease course. Secondary outcomes included long-term outcomes such as disease remission and the development of comorbidities, particularly calcinosis. Clinical remission was defined as the absence of active myositis and skin rashes. The absence of active myositis was determined with normal muscle strength and serum muscle enzyme levels. Active and inactive diseases were described according to the PRINTO criteria for clinically inactive disease in juvenile dermatomyositis [18].

### Statistical analysis

Descriptive statistics were used to describe the data. For categorical variables, frequencies and percentages were reported. Differences between groups were analyzed using Pearson’s χ2 tests (or Fisher’s exact tests for expected cells < 5). For continuous variables, mean and standard deviation were used to present the data while analysis were performed using Student’s t-tests. For continuous variables not normally distributed, they were summarized using median and interquartile range and analysis performed using Wilcoxon-Mann-Whitney tests. An a *priori* two-tailed level of significance was set at 0.05. Statistical analyses were conducted using STATA version 13.1 (STATA Corporation, College Station, TX, USA).

### Ethical considerations

Ethical approval was obtained from the institutional review boards of all participating centers (MERC #3430). Patient confidentiality was strictly maintained throughout the study. This study adhered to the principles of the Declaration of Helsinki. Written informed consent was waived due to the retrospective nature of the study, and all data were anonymized before analysis.

## Results

### Patient characteristics

During the study period, thirty Omani children with JDM were included. The cohort of patients was distributed across various regions, with the majority originating from Muscat (40%; *n* = 12), followed by the Al-Batinah region (27%; *n* = 8) reflecting population density and healthcare accessibility in these areas. Other regions represented included Al-Sharqiyah (13%; *n* = 4), Al-Dhahirah (6.7%; *n* = 2), and Al-Dakhiliyah (6.7%; *n* = 2). Fifteen (50%) of the patients were female, resulting in a female-to-male ratio of 1:1.

The median age at disease onset was 6.78 years (absolute range: 2–13 years), while the median age at diagnosis was 7.3 years (absolute range: 2–13). Consequently, the median duration from symptom onset to diagnosis was 8.4 months, with a wide absolute range of 0–23 months, reflecting significant variability in the time taken to diagnose the condition. The median follow-up period for these patients was 4 years (absolute range: 1 month-16 years).

Among the 30 patients, 15 (50%) had creatine kinase (CK) levels below the upper normal limit (< 308 U/L). Of these, 6 patients (40%) experienced a diagnostic delay exceeding 6 months. The median delay among this subgroup was 15 months (12–23 months). Despite a relatively normal CK level, classic clinical features were common: heliotrope rash was present in 5 (83%), Gottron’s papules in 4 (67%), and proximal muscle weakness in all 6 patients (100%). Notably, CK levels declined further following initiation of immunosuppressive therapy in these patients, suggesting that their initial “normal” values may have been relatively elevated for their disease state. This pattern may reflect chronic muscle damage, reduced muscle mass, or a subacute disease course that masked the biochemical evidence of active myositis.

### Diagnostic features

The clinical features of JDM cohort is shown in Table 1. Diagnostic clinical JDM features including proximal muscle weakness and cutaneous manifestations were documented in all 30 patients. The classic JDM rashes, heliotrope rash and Gottron’s papules, were the most highly prevalent cutaneous manifestations, affecting 25 (83%) and 23 (77%) patients, respectively. The median values of muscle enzymes and inflammatory markers are shown in Table 2. Muscle enzymes were elevated in majority of cases. CK was elevated (> 308 U/L) in 50% (15/30); AST was elevated (> 41 U/L) in 70% (21/30) and LDH were elevated (> 300 U/L) in 67% (20/30) of the patients. These patients have either MRI, muscle biopsy and/or EMG to support the diagnosis of JDM. MRI was conducted in 27 patients, with 19 (70%) patients demonstrating evidence of active muscle inflammation, including hyperintense signals on T2-weighted or STIR images, consistent with muscle edema, and gadolinium-enhanced T1-weighted images showing contrast uptake in inflamed areas. Muscle biopsy was performed in 9 (30%) patients and revealed characteristic features of juvenile dermatomyositis, including perivascular atrophy, fiber degeneration and regeneration, and perivascular inflammation. Muscle biopsies were obtained based on clinical indications such as atypical presentation, inconclusive MRI or laboratory findings, or to rule out alternative diagnoses. EMG was conducted in 8 (27%) patients showing characteristic features consistent with myositis. The use of EMG and muscle biopsy was limited in our cohort despite their diagnostic value in JDM due to their invasive nature, need for patient cooperation, and risk of sampling error from patchy muscle involvement. Both procedures also require specialist expertise for accurate interpretation. These limitations support the use of MRI as a more non-invasive, and practical alternative.

**Table 1:** Clinical characteristics of JDM cohort (*n* = 30)

Clinical features	*n* (%)
Muscle weakness	28 (93%)
Proximal	28 (93%)
Distal	5 (17%)
Truncal	5 (17%)
Dysphagia	2 (6.7%)
Dysphonia	0
Gower sign	15 (50%)
Rash	25 (83%)
Heloiotrope	25 (83%)
Gottron	23 (77%)
Malar Erythema	13 (43%)
Capillary loop changes	11 (37%)
Calcinosis	8 (27%)
Skin ulcer	4 (13%)
Photosensitivity	4 (13%)
Lipodsytrophy	2 (6.7%)
Constitutional sx	29 (97)
Fatigue	25 (83%)
Weight Loss	8 (27%)
Fever	7 (23%)
Arthralgia/Arthritis	23 (77%)
GI (Abdominal pain)	5 (17%)
Lung	1 (3.3%)
Cardiac	0

**Table 2:** Muscle enzyme values in JDM cohort *n* = 30

Investigation	Reference	Median (IQR)	No (%)
CK	26–309 U/L	265 (74-1485)	15 (50)
LDH	120–300 U/L	449 (328–670)	20 (67)
ALT	0–41 U/L	45 (17–76)	15 (50)
AST	0–41 U/L	86 (30–173)	21 (70)

IQR, interquartile range

### Myositis antibodies

Myositis-specific antibodies (MSA) and myositis-associated antibodies (MAA) were tested in 26 patients. Among the MSA, the following were positive: NXP2 in 3 patients, Anti-Mi-2 in 1 patient, Anti-TIF1 in 2 patients, and MDA5 in 1 patient. Among the MAA, the following were positive: Anti-SSA in 6 patients, Anti-Scl-100 in 1 patient, and Anti-PmScl in 1 patient.

In our cohort, myositis-specific antibodies (MSA) showed expected correlations with their known clinical associations. Among patients with NXP2, 2 out of 3 developed calcinosis, while both Anti-TIF1-positive patients had severe skin involvement, consistent with its recognized phenotype. The patient with Anti-Mi-2 experienced rapid remission, reflecting its typically favorable prognosis. Finally, the MDA5-positive patient did not develop lung involvement, despite reported association with this antibody.

Myositis-associated antibodies (MAA) presented more variable clinical patterns. Half (3/6) of patients with SSA antibodies exhibited overlap features, including characteristics of mixed connective tissue disease (MCTD). However, none of the patients with Anti-Scl-70 or Anti-PmScl showed clinical features typically linked to systemic sclerosis or other overlap syndromes.

### Treatment

**Fig. 1:**
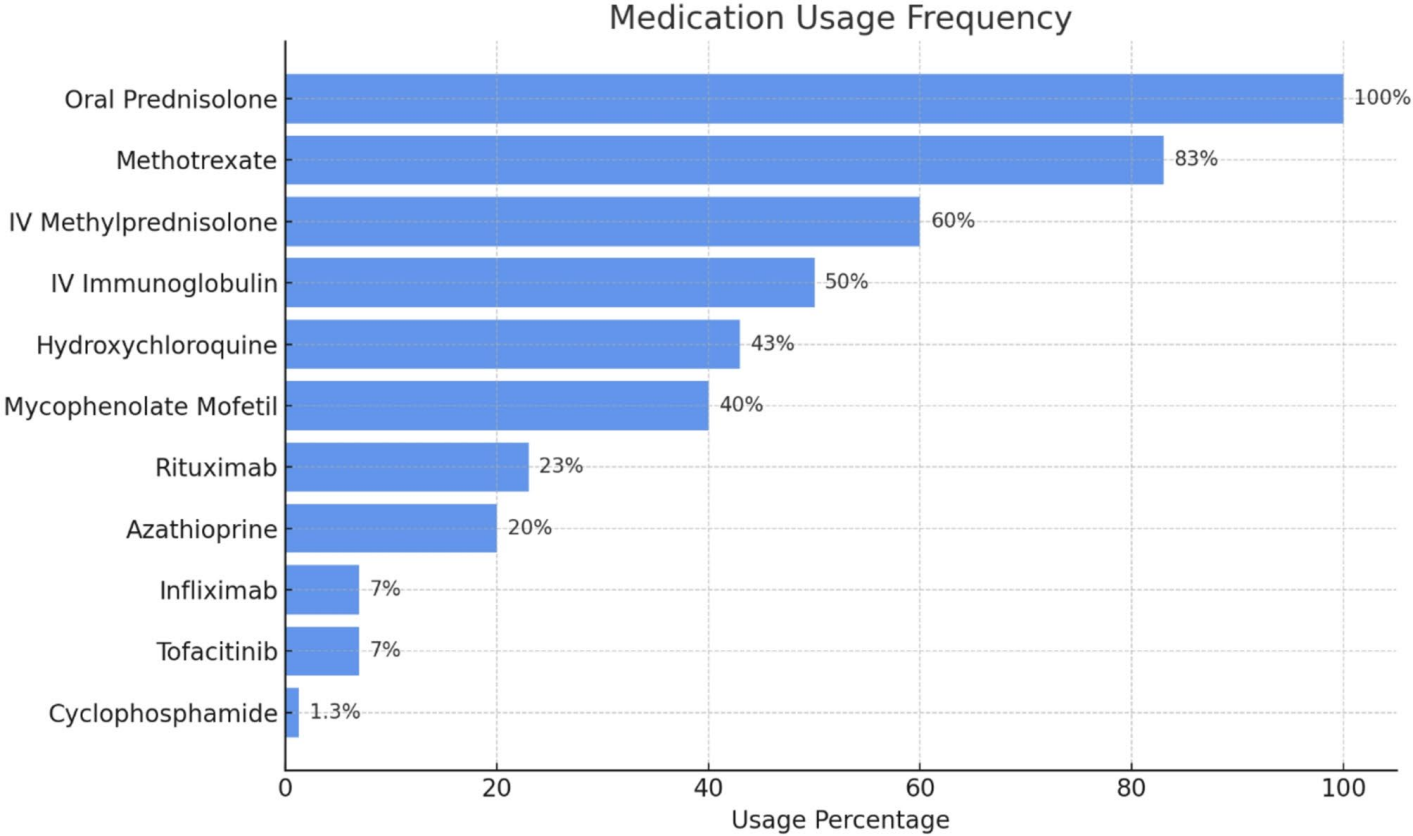
Treatment modalities in JDM cohort *n* = 30

Treatment modalities for JDM in our cohort is illustrated in Fig. 1. In our cohort, patients presenting with severe disease were typically initiated on triple therapy comprising intravenous methylprednisolone (IVMP), methotrexate (MTX), and intravenous immunoglobulin (IVIG). The IVIG regimen followed the CARRA (Childhood Arthritis and Rheumatology Research Alliance) consensus recommendations, typically administered at a dose of 2 g/kg over 2 days monthly, continued for at least 6 months depending on clinical response [25]. Escalation to additional DMARDs, was considered in cases where patients experienced recurrence or refractory cutaneous or muscular disease, particularly during corticosteroid tapering. Treatment decisions were individualized and based on clinical response, disease trajectory, and tolerability.

All patients received oral corticosteroids (30/30, 100%), and 18 (60%) were treated with IVMP. MTX was used in 25 patients (83%), while IVIG was administered in 15 (50%). Other immunosuppressants included mycophenolate mofetil in 12 patients (40%), hydroxychloroquine in 13 (43%), azathioprine in 6 (20%) and cyclophosphamide 1 (3.3%) patient. Biologic agents were also used including, rituximab in 7 patients (23%), infliximab in 2 (7%), and tofacitinib in 2 (7%) patients. For calcinosis management, pamidronate was given to 3 (10%) and diltiazem to 2 (7%) patients.

### Disease course and outcome

At the last clinical visit, 18 (60%) patients met criteria for clinical remission, defined by the absence of muscle weakness and skin manifestations, along with normal muscle enzyme levels, based on PRINTO criteria [18]. Remission was defined regardless of whether patients were still receiving maintenance therapy. Additionally, 4 patients developed overlap or MCTD, 7 were lost to follow-up, 4 transitioned to adult care, and 2 died due to sepsis. Consistent with known patterns of disease progression, 15 patients (50%) exhibited a monophasic course, while the remaining patients followed a polyphasic or continuous disease course.

## Discussion

In this study, we report the clinical characteristics, diagnostic features, treatment patterns, and outcomes of JDM in Oman. Our findings contribute to the understanding of JDM in the Middle Eastern population, highlighting both similarities and differences with global cohorts as demonstrated in Table 3.

**Table 3:** Comparison of epidemiological clinical treatment and outcome of JDM cohort across global cohorts

		Oman	Arab 17	Canada 19	CARRA 20	Turkey 21	Thai 22	Africa 23	India 24
Patient no		30	92	172	119	50	30	25	86
Female no (%)		15 (50)	58 (63)	110 (64)	76(63.4)	35 (70)	22 (73)	16 (64)	46 (53.5)
Age diagnosis		7.3 ± 3.5	6.6 ± 3.0	8.5 ± 4.3	8 (4-11.5)	6.6 ± 4.1	5.1 (2.6–14.8)	7.95 (3.4–9.7)	10.0 (5.7–14.5)
Age at onset		6.8 ± 3.5	6.0 ± 3.0		7 (3.5–7.5)	6.1 ± 4.1	4.1 (0.6–14.3)	6.75 (2-9.7)	
Period to diagnosis		8.4 m (0–108 m)	1.5 ± 5.6	3.0 months (IQR 1.6–6.2 months)	3.0 m IQR 1–6.5 m)	5.2 ± 2.5 months– no calcinosis 7.1 ± 2.3 months with calcinosis	6.5 (0.3–84 months)	4 m (0.5–84 m)	6 m (4–18 m)
Follow up		4.9 ± 4.7	5.0 ± 4.4	5.57 ± 2.3		6.2 ± 4.1	3.1 (0–18)	50 month	30 month
	Course type (%)								
Monocyclic		50	37	29	40	NA	30	NA	47.7
Continuous & Persistent		65	63	38	60	NA	50	NA	20.9
	Clinical Features %								
Constitutional symptoms		97	40	NA	60	NA	28.6	NA	47.6
Weakness		93	94	85.5	72.3	90	90	100	91.8
Gottron’s		77	78	78.5	75.6	96	83.3	76	69.7
Heliotrope		83	70	66.3	53.8	100	70	80	61.6
Arthritis		83	49	42	27.7	2	22.2	28	19.7
Dysphagia		6.7	15	25	17.6	2	6.9	20	27.9
Cutaneous ulcer		13	8	10	11	6	20.7	44	26.7
Pneumonitis		3	11	2.3	1%	2	3.4	16	1.2
Gut vasculitis		17	13	23	NA	NA	NA	12	NA
	Treatment: %								
IVMP		60	60	31	NA	NA	48.1	40	NA
Oral Pred		100	100	90	100	100	96.3	100	100
Methotrexate		83	86	64	NA	96	74	64	83.7
Cyclosoporin		3	10	1.2	NA	48	3.7	0	NA
MMF		40	NA	0	1	0	18.5	12	1.2
IVIG		50	33	15.7	2.3	18	11.1	40	2.3
Hydroxychloroquine		43	17	4.7	NA	NA	66.7	32	NA
Cyclophosphomide		1.3	7.6	2.3	NA	10	10	12	8.1
Biologics		10	10.8	0	7	4	0	16	6.9
Pamidronate		10	13	NA	NA	34	0	NA	NA
	Outcome								
Remission %		60	Most 16 skin changes 12 muscle weakness	NA	NA	NA	30	40	47.7
Calcinosis (%)		27	29.3	23	3.4	38	21.4	44	18.2
Death (n)		2	2	0	NA	0	10	8	2

Over a 16-year period, 30 patients were diagnosed with JDM in pediatric rheumatology centers across Oman. According to the 2020 Census by the Oman National Centre for Statistics and Information, the estimated number of children < 14 years of age is approximately 1.1 million. This yields an estimated annual incidence of JDM of 1.7 per million children, which is consistent with the global average of 2–4 per million [1].

The gender distribution in our cohort was equal between males and females, contrasting with global female predominance observed in other studies [17, 19–24]. The mean age at diagnosis (7.3 ± 3.5 years) was comparable to other cohorts, including those from the Arab region, Turkey, and South Africa [17, 18, 23]. The youngest age at diagnosis was observed in the Thai patients (5.1 years, range 2.6–14.8 years), whereas the Indian cohort had the oldest age at diagnosis (10.0 years, range 5.7–14.5 years) [22, 24].

While the mean time from disease onset to diagnosis was similar across cohorts, the wide range in diagnostic delays is concerning [17, 19–24]. In Oman, in certain cases, the delay extended up to 23 months, mirroring trends in South Africa and India [23, 24]. These prolonged delays highlight regional disparities in JDM recognition, likely due to differences in healthcare access, disease awareness, and diagnostic capabilities.

While delayed referral was noted, particularly those presenting with normal muscle enzyme levels despite classic clinical features, our sample size was too small to draw statistically meaningful conclusions about the impact of late diagnosis on long-term outcomes. Similarly, although we had some patients from more remote geographic regions (e.g., Al-Sharqiyah, Al-Dhahirah), we did not observe a any trend linking geographic origin or referral delay with worse clinical outcomes, including remission rates or development of complications such as calcinosis.

The clinical manifestations in our cohort aligned with global trends, with universal proximal muscle weakness and cutaneous involvement, reinforcing their clinical importance in JDM diagnosis [17, 19–24]. Notably, arthritis is more frequent in our cohort (77%) compared to other groups, possibly reflecting variations in clinical evaluation. Whereas dysphagia (27.9%) and cutaneous ulcers (26.7%) are more frequently observed in the Indian cohort, indicating a more severe disease phenotype with greater muscle and vascular involvement [24].

The treatment approach in our cohort, including corticosteroids, MTX, and IVIG, aligns with international guidelines and global practices [25]. The use of biologics (rituximab) was most frequent in South Africa, followed by Arab cohorts, including Oman (Table 3). This may be attributed to delays in diagnosis necessitating advanced immunosuppression. Furthermore, variations in physician preferences and easier access to biologics compared to other regions may have facilitated earlier adoption of biologics when standard therapies were insufficient.

JDM follows three distinct clinical courses: monocyclic, chronic persistent, and polycyclic. The distribution of these disease patterns varies across populations. Studies from Hungary and India report a higher proportion of patients with a monocyclic course (59–73%), suggesting a greater likelihood of early disease resolution [26, 27]. In contrast, research from Europe and North America indicates a greater likelihood of patients experience a chronic or polycyclic disease course (57–93%) [21]. This variation may be attributed to genetic or environmental influences on disease severity that my contribute to these patterns.

The remission rate of our cohort was 57%, comparable to other JDM cohorts (33–60%), with several factors contributing to these variations [26, 27]. Differences in remission criteria impact reported rates, as some define remission as medication-free inactive disease, while others include controlled disease on therapy. Variability in assessment tools (e.g., PRINTO, IMACS) and treatment tapering strategies also affect outcomes. Variability in the timing of therapy initiation across cohorts, often due to delayed diagnosis, may influence remission rates, as prolonged uncontrolled disease can lead to refractory disease.

The incidence of calcinosis in our cohort (27%), is comparable with global reports ranging from 4 to 44% as shown in Table 3. The striking difference in calcinosis incidence between the South African (44%) and CARRA (4%) cohorts can be attributed to several factors [20, 23]. The South African cohort had longer diagnostic delays (median 6 months, with some cases up to 7 years) compared to CARRA (median 3 months). While CARRA patients were managed using standardized Consensus Treatment Plans (CTPs), ensuring structured and early intervention, the South African cohort had a varied treatment approach. Chronic disease activity at follow-up was similar in both cohorts (60%), but South Africa’s longer follow-up (50 months) likely increased calcinosis detection. In contrast, CARRA tested for myositis-specific antibodies, including anti-NXP2, while South Africa did not, leaving autoantibody influence uncertain. Furthermore, socioeconomic factors, including limited healthcare access may have contributed to more severe disease and complications in the South African cohort.

Mortality in JDM has significantly declined over the past decades, primarily due to advancements in immunosuppressive therapies, particularly corticosteroids. Historically, JDM-related mortality exceeded 30% but decreased to approximately 10% with the introduction of corticosteroid therapy [26, 27]. However, global mortality estimates of JDM remain between 5 and 8% [26, 27]. In our cohort, 2 patients died due to sepsis. Both patients were males with chronic persistent and refractory JDM. The first died at age 15, following a 4-year disease course complicated by JDM-MCTD overlap and transverse myelitis. He had been treated with corticosteroids, IVIG, MMF, and rituximab, and was admitted with multiorgan failure secondary to *Pseudomonas aeruginosa* sepsis. The second, aged 13 with one year of refractory disease, had received IVMP, methotrexate, IVIG, and MMF. He developed a chest infection complicated by *Aspergillus* pneumonia and *Pseudomonas* bacteremia, culminating in multiorgan dysfunction. These cases underscore the heightened risk of severe infections and adverse outcomes in patients with prolonged, treatment-resistant JDM receiving intensive immunosuppressive therapy.

This study has several limitations inherent to its retrospective design, which may affect data accuracy and generalizability. Incomplete or inconsistent medical records could have introduced information bias, potentially leading to underreporting of clinical features and treatment outcomes. Additionally, the relatively small sample size (*n* = 30) limits the ability to fully capture the clinical spectrum of JDM in Oman and reduces the generalizability of our findings. The absence of standardized investigations, including MSA assessments across all patients restricts the ability to analyze potential serological correlations with disease phenotype and outcomes. Furthermore, variability in follow-up duration may have also influenced reported remission rates and disease course classification. Moreover, the assessment of muscle strength using standardized tools like the Childhood Myositis Assessment Scale (CMAS) and Manual Muscle Testing of eight muscle groups (MMT8) was applied inconsistently across the patients and not systematically documented, precluding their inclusion in the analysis. These limitations underscore the need for prospective, multicenter studies with standardized diagnostic and treatment protocols to enhance the understanding of JDM’s clinical course and improve patient outcomes.

## Conclusion

This study provides a comprehensive overview of juvenile dermatomyositis in Oman, highlighting diagnostic delays, particularly in patients with typical symptoms but normal muscle enzymes. Autoantibody profiles mirrored known phenotype associations, and remission rates were broadly consistent with international cohorts. Calcinosis remained a notable complication, and the frequent use of biologics may reflect local treatment practices. These findings underscore the importance of early diagnosis, sustained follow-up, and individualized management to improve long-term outcomes.

This national cohort study provides insights into the clinical patterns, serologic profiles, and outcomes of JDM in Oman. A key finding was diagnostic delay, often in patients presenting with classic cutaneous and muscular symptoms but relatively normal CK levels. This suggests a critical gap in physician awareness, as a relative normal or near-normal CK may not exclude disease activity. Autoantibody testing revealed phenotype-specific patterns consistent with international data, such as the association of NXP2 with calcinosis. While most patients responded to conventional immunosuppressive therapies, the need for biologic agents in 37% of may reflect differences in therapeutic strategies. With 60% of patients achieving clinical remission and a 27% calcinosis rate, this study highlights the clinical relevance of timely diagnosis, improved disease recognition—including relatively non-elevated muscle enzymes—and the importance of individualized, long-term management strategies to optimize outcomes in pediatric JDM.

## Data Availability

Research data can be available upon request from authors.
